# The prospective and concurrent effect of exercise on health related quality of life in older adults over a 3 year period

**DOI:** 10.1186/s12955-018-0843-9

**Published:** 2018-01-16

**Authors:** Helen-Maria Vasiliadis, Mathieu F. Bélanger

**Affiliations:** 1 Faculty of Medicine and Health Sciences, Université de Sherbrooke, Sherbrooke, Quebec Canada; 2 Charles-Le Moyne Hospital Research Center, Greenfield Park, Quebec Canada; 3 Centre de formation médicale du Nouveau-Brunswick, Moncton, New Brunswick Canada; 4 Vitalité Health Network, Moncton, New Brunswick Canada; 5 Université de Sherbrooke, Centre de recherche – Hôpital Charles-Le Moyne, Campus Longueuil, 150 Place Charles-Le Moyne, Longueuil, (QC) J4K 0A8 Canada

**Keywords:** Physical activity, Health related quality of life, Older adults

## Abstract

**Background:**

It is not clear whether socioeconomic status influences health outcomes among older adults through its effect on physical activity. The aim of this study was to assess the effect of sex and neighborhood socio-economic status on the change in health related quality of life (HR-QOL) as a function of physical activity over a three-year period.

**Methods:**

This cohort study included French-speaking community-dwelling older adults recruited in primary care practices in the province of Quebec and participating in the ‘*Étude sur la Santé des Ainés’* (ESA)-Services study on the health of the elderly. Primary care practices were recruited through participating general practitioners (GPs) working full-time in the health administrative region. A stratified sample was comprised of various types of primary care practices (family medicine group, local community health services centers, primary care practices with less than 3 GPs, and with at least 3 GPs). In this study sample, 967 participants with scores ≥26 on the Mini Mental State Examination (MMSE) were included and followed for 3 years to study HR-QOL as a function of reported exercise at baseline and follow-up, controlling for study variables. Analyses were also carried out to study the effect of change in reported exercise at follow-up with respect to baseline and categorised as follows: no change, decrease in exercise and increase in exercise. The interaction terms area of residence socio-economic status*exercise and sex*exercise, were tested.

**Results:**

Exercise at baseline did not significantly predict HR-QOL at follow-up when adjusting for all other study variables. Exercise at follow-up was cross-sectionally associated with follow-up HR-QOL. Participants reporting never exercising and those reporting a decrease in exercise reported a lower HR-QOL at follow-up, when controlling for all other study variables. There was no interaction between exercise and sex and socioeconomic status.

**Conclusions:**

For healthy ageing, maintaining and increasing physical activity throughout the years is necessary for improved HR-QOL. Past physical activity does not confer protection against future decline of HR-QOL. Future research should focus on potential moderating and mediating psycho-social barriers associated with exercising in older age populations.

## Background

By 2050, the population of individuals aged 65 years and older will double worldwide and make up 30% of the Canadian population [1]. Chronic disorders in the older adult population accounts for a quarter of global disease burden [2]. To prevent challenges accompanying aging of the population, a focus on health policies aimed at promoting healthy living has taken place in Canada and worldwide [3]. Reviews have underlined the importance of increasing physical activity to reduce the risk of mortality, cardiovascular disease, diabetes, cancer, dementia and depression in older adults [4–6]. Other systematic reviews also underlined the importance of physical activity as a determinant of health related quality of life (HR-QOL), mental health, vitality, and general health [7, 8]. Guidelines for promoting healthy ageing recommend “at least 150 minutes of moderate- to vigorous-intensity aerobic physical activity per week” [3, 9].

Data from the 2012-2013 Canadian Health Measures Survey showed that older adults aged 60 to 79 years were less likely to be active than their younger counterparts; only 12% met recommended guidelines for physical activity [10]. These data also indicate that, on average, males are significantly more active than females. However, sex differences in health outcomes related to changes in physical activity in older adults are inconclusive [11–13]. An earlier report also showed that, in Canada, respondents in the highest income quintile were more likely to report active or moderately active leisure-time physical activity as compared to those in the four lowest income quintiles and this while controlling for other socio-demographic factors [14]. Others have also shown positive associations between education and income, as well as neighborhood factors such as wealth, aesthetics and improved settings for physical activity [15–17]. It is therefore possible that socioeconomic status influences health outcomes among older adults through its effect on physical activity.

Although a recent German socioeconomic panel study on older adults did not show a synergistic effect of socioeconomic status, measured by education and income, on physical activity and self-perceived health in the following year, this study was limited by having only a short follow-up period (1 year) and only accounting for age, migration, region and type of residence in the analyses [18]. To ascertain whether socioeconomic status influences the effect of physical activity on health status among older adults, it appears important to take consideration of potentially confounding factors identified in the literature including sex, health status, lifestyle habits, social support, stressful life events and neighborhood socioeconomic factors [19, 20]. It is also important to investigate associations over a longer period of time. To help inform health policies on improving healthy ageing, the aim of this study was to assess the effect of sex and neighborhood socio-economic status on the change in HR-QOL as a function of physical activity over a three-year period in a sample of community living older adults.

## Methods

The sample consisted of older adults aged ≥65 years recruited in the “*Étude sur la Santé des Aînés*” (ESA)-Services study (2011-2013). Participants were recruited while they were in a waiting room of one of the general practice clinics in the Montérégie health administrative region, which is one of the most populated health regions in the province of Quebec, Canada. Clinics were recruited via general practitioners (GPs) working in the region full time (≥4 days/week). The sample included stratification according to type of primary care practice, which included family medicine groups, local community health services centers, primary care practices with less than 3 GPs, primary care practices with at least 3 GPs). Over 60% of eligible physicians (*n* = 744) participated in the study. Physician participation did not depend on gender or type of practice (*p* = 0.09). For each GP recruited, pamphlets explaining the objectives and inviting patients to participate in the study were placed in the waiting room of their practices. Overall, 249 practices recruited an average of 7.3 patients each. A detailed description of the recruitment and methodology of the ESA-Services study has been published elsewhere [21].

Briefly, individuals interested in participating were contacted within 30 days of the visit and were scheduled an at home interview if they were French-speaking. Informed consent was obtained at the beginning of interviews, which were carried out in an isolated area of the home to limit any reporting bias if there was another person present. In order to minimise information bias associated with cognitive impairment, individuals in the 10th percentile of the Mini Mental State Examination (score < 26) were excluded from the analyses [22]. Among the 1627 remaining individuals who had given their consent to be contacted for a follow-up study, we were able to contact by telephone and schedule interviews for 967 individuals 3 years later. Interviews were led by research assistants who were trained to administer measures according to a standardised protocol and to recognise and implement a pace of questionnaire administration that was comfortable for participants. Participants were compensated $15CDN per visit with a check mailed to their home within a month of the interview. The study was approved by the University of Sherbrooke Institute of Geriatrics ethics committee.

### Measures

#### Health related quality of life

HR-QOL was based on 29 questions relating to the presence of difficulties in the following 5 domains: mobility, self-care, usual activities, pain and anxiety/depression, which are similar to those included in the EuroQol EQ-5D questionnaire on health [23].

The EuroQol EQ-5D is a generic health related quality of life instrument which elicits the subject’s health state on 5 dimensions attributed to pain, mobility, self-care, usual activities and anxiety/depression. The EuroQol EQ-5D-3 L includes 3 possible levels indicating no problems, some problems or extreme problems. A preference weight elicited by the general population can then be applied to each attribute and the final score is used to represent the subject’s current health state [24, 25]. To reduce the length of interviews and avoid redundancy in questions, only a sub-sample of the EuroQol EQ-5D-3 L was used. As documented elsewhere, available data still enabled us to reconstitute answers for each of the 5 domains covered [24, 25]. The questions pertaining to activities and instrumental activities of daily living, pain and anxiety or depression ranged from no problem to extreme problem.

The domain ‘mobility’ was based on the level of difficulty reported with either walking indoors, walking outdoors or using stairs which was categorized as: no problems walking, some problems in walking about, confined to bed. For example, the EQ-5D question on mobility asks the respondent which statement best describes their health today. Possible answers included, ‘I have no problem walking about’, ‘I have some problems in walking about’ and ‘I am confined to bed’. The ESA-Services questionnaire included the following 3 questions on mobility: ‘Do you have difficulty walking indoors?’, Do you have difficulty walking outdoors?, ‘Do you have difficulty using stairs?’ Possible answers included: ‘Circulates alone (with or without a cane or other aide)’; ‘Circulates with some difficulty’, ‘Circulates with the help or guidance of another person, needs to be supervised’, ‘Does not walk’, ‘Does not use stairs’. Respondents were then categorized as ‘I have no problem in walking about’ if they answered ‘circulates alone’ and ‘no problem in going up or down the stairs’; categorized in the ‘I have some problem in walking about’ if they answered ‘circulates with some difficulty or help’; while all others were categorized in the highest level of disability, ‘I am confined to bed’.

The domain ‘self-care’ was based on the level of difficulty reported in feeding, bathing, dressing and toileting which was categorized as: no problem with any of the activities or some problems if they reported being unable to perform at least one self-care activity. The domain ‘usual activity’ was based on the level of difficulty with housekeeping, laundry, food preparation and shopping, which was categorized as no problem with any of these activities or some problems if they reported being unable to perform at least one of these activities.

Pain or discomfort (no, moderate, extreme pain or discomfort) was directly assessed with one question, as presented in the EuroQol EQ-5D-3 L. The domain related to anxiety and depression was based on scores obtained on the 7-item Generalized Anxiety Disorder (GAD) Scale (scores ranging between 0 and 21) [26] and the 10-item Kessler Psychological Distress Scale (K-10) (scores ranging between 10 and 50) [27]. GAD scores ranging from 0 to 9; 10 to 14 and ≥15 were categorized as not, moderately and extremely anxious, respectively. K-10 scores ranging 10 to 24; 25 to 29 and ≥30 were categorized as unlikely, moderately and extremely depressed, respectively [28–30]. Participants were then categorized in the corresponding EQ-5D-3 L category as follows: ‘I am extremely anxious or depressed’ if their score indicated extremely likely to be depressed or to have anxiety; ‘I am moderately anxious or depressed’ if they scored as moderately likely; and ‘I am not anxious or depressed’ if their score indicated unlikely depressed and anxious, on the K-10 and GAD-7.

Each domain was therefore categorized according to level of difficulty described in the EQ-5D-3 L: level 1, indicating no problem; level 2, indicating some problems and level 3, indicating extreme problems. These produced a specific health state for each individual. The EQ-5D health states, were then converted into a summary index ranging from 0 (death) to 1 (perfect health) as described by Rabin et al., (2011), which gives a formula that attaches values (also called weights) to each of the levels in each dimension [31]. Since the original EQ-5D-3 L was not used, we verified the concordance between the calculated summary index with respondent scores on the EuroQOL visual analog scale (ranging from 0 to 100; worst imaginable health state to best imaginable health state). The results showed that the scores were highly correlated (*r* = 0.36; *p* < 0.0001). The EQ-5D-3 L in older adults has shown good reliability with intraclass correlation coefficient reaching 0.79 and good construct validity with the of the SF-12 physical health component scale reaching 0.60 [32].

#### Physical activity

Physical activity was assessed with the following question*: “How many times a week do you exercise for more than 20 minutes (for example, walking at a rapid pace)”.* Prior research has shown that similar single self-reported items are acceptable and perform just as well as other questionnaire-based measures of physical activity [13, 33].

#### Other study variables of interest

Health status was measured with the self-reported presence of a chronic disorder diagnosed by a physician which participants could report with on a list including arthritis, heart and cardiovascular diseases, diabetes and endocrine system disorders, digestive system, and kidney, liver and eye disorders [34]. The presence of multimorbidity (yes/no) was based on the presence of at least 3 chronic conditions and more [35].

The presence of depression (major or minor) was assessed following DSM-5 guidelines [36]. Individuals who reported, in the past 6 months, having either a depressed mood or loss of interest or pleasure in usual daily activities nearly every day and most of the day during at least 2 consecutive weeks, and a total of at least three out of the nine symptoms of depression, with impairment in at least one area of social functioning were categorized as having depression. Those who were told by their doctor that these symptoms were due to a physical disease or medication were categorized as not having depression.

The presence of social support was based on questions from previous health surveys in Quebec which included the presence of someone (1) to talk about various problems, (2) who could provide instrumental help, and (3) who could provide emotional support (ISQ 1998).

The presence of daily hassles as stressors was measured with the adapted French version of Kanner’s [37] Daily Hassle Scale for older adults, which has shown good reliability (test–retest reliability coefficients of 0.79 and a Cronbach *a* of 0.90). The 22- item Likert scale includes questions on daily hassles relevant to older adults with scores ranging from ‘not at all to extremely stressful’. The number of daily hassles were based on the addition of items scored as ‘a little to extremely stressful’.

Area of residence socio-economic status was characterized by the validated Pampalon indices [38, 39] of material and social deprivation which are based on the following six geographic indicators: the proportion of people aged 15 years and over without a certificate or high school diploma; the proportion of persons 15 years and over that are employed; the average income of persons 15 years and older; the proportion of people aged 15 years and over living alone in their homes; the proportion of people aged 15 years and over who are separated, divorced or widowed; the proportion of single-parent families. These indicators can be summarized into one index on a scale of 1 to 5, representing the most advantaged to the most disadvantaged quintiles [highest socioeconomic status, medium high, medium, medium-low, low] [40]. This variable was dichotomized to identify area of residence with high social or material deprivation (quintiles 4 and 5) versus low to medium deprivation (quintiles 1-3) [41, 42].

Lifestyle habits considered included smoking status (current smoker yes/no) and frequency of consuming at least one alcoholic drink at least twice a week every week in past 6 months (yes/no). Additional study variables considered in the analyses as possible confounding factors included sex, age (65 – 74 years versus 75 years and over), yearly income (< 55,000 or ≥ 55,000$), education (primary versus ≥ high school).

### Analyses

Multivariate regression analyses were carried out to study HR-QOL as a function of exercise at baseline and follow-up, controlling for study variables. Additional analyses were also carried out to study the effect of change in reported exercise at follow-up with respect to baseline and categorised as follows: never exercised (no exercise at baseline and follow-up), always exercised, decrease in exercise and increase in exercise. The interaction terms tested [area of residence socio-economic status*exercise] and [sex*exercise] were not significant (*p* = 0.45; *p* = 0.97, respectively) and as such analyses were not stratified by socio-economic status or sex. A forward stepwise regression analysis, with a significance entry level of 0.05, while forcing baseline and follow-up exercise and HR-QOL at baseline into the model, was also carried out to identify study variables with a significant effect on HR-QOL at follow-up. Missing data for area of residence and HR-QOL were imputed by maximizing internal consistency [43]. Unadjusted and adjusted estimates with 95% CI are presented. Analyses were carried out with the REG procedure in SAS version 9.4.

## Results

On average, individuals lost to follow-up reported a lower HR-QOL (*p* = 0.008) and a lower number of times of exercise in a week (*p* < 0.001) at baseline. Individuals lost to follow-up were more likely to be female (*p* = 0.02), aged between 65 and 74 years (*p* = 0.01), to have finished primary school only (*p* = 0.002), to report a high income (*p* = 0.004), be current smokers (p < 0.001) but less likely to drink alcohol on a weekly basis (*p* = 0.0003). There was no significant difference between individuals retained and lost to follow-up with respect to education, income, marital status, social support, daily hassles, neighbourhood deprivation, number of chronic conditions and depression.

Respondents exercised on average close to 4 times a week (Table 1). Exercise at baseline was a significant predictor of HR-QOL 3 years later in the unadjusted model and model controlling for exercise at follow-up and baseline HR-QOL (Table 2). However, exercise at baseline did not significantly predict HR-QOL at follow-up when adjusting for all other study variables. We carried out further analyses to understand which study variable had the most significant effect on the association between baseline exercise and HR-QOL at follow-up. This analysis showed that current smoking, the presence of more than 2 chronic conditions and sex significantly explained the variability in HR-QOL at follow-up that was previously explained by baseline exercise.

**Table 1:** Characteristics of study sample retained for analyses *N* = 969

	Baseline		Follow-up	
Variables	Mean	(SD)	Mean	(SD)
Health related quality of life (− 0.594, worst HR-QOL to 1, best HR-QOL)	0.81	0.19	0.70	0.11
# Times a week exercising for more than 20 min	3.86	4.19	4.00	4.76
	N	%	N	%
Sex				
Males	429	44%	429	44%
Females	540	56%	540	56%
Age				
65 – 74 years	657	68%	504	52%
≥ 75 years	312	32%	465	48%
Marital status				
Married	635	66%	619	64%
Not Married	334	34%	350	36%
Education				
Primary level	79	8%	106	11%
High School and post-secondary	890	92%	863	89%
Yearly Household Income				
< 55,000$	957	99%	947	98%
≥ 55,000$	12	1%	22	2%
Area of residence				
High social or material deprivation	403	42%	558	58%
Low/Medium social or material deprivation	566	58%	411	42%
Current smoker				
No	901	93%	909	94%
Yes	68	7%	60	6%
At least one alcoholic drink, ≥ twice a week, every week in last 6 months				
No	616	64%	662	68%
Yes	353	36%	307	32%
# of chronic physical disorders				
0-2	322	33%	319	33%
≥ 3	647	67%	650	67%
Depression				
No	914	94%	943	97%
Yes	55	6%	26	3%
Social support				
0-2	120	12%	135	14%
3	849	88%	834	86%
Number of Daily Hassles				
0-4	536	55%	468	48%
4+	433	45%	501	52%

**Table 2:** Prospective association between exercise frequency and health related quality of life at follow-up, 3 years later

	Health-related quality of life (EQ 5D scores)		
	Unadjusted	Adjusted*	Adjusted *
		Beta estimates (95% CI)*	
# Times a week exercising at baseline for more than 20 minutes	*0.003 (0.001, 0.005)ª*	*0.002 (0.0004, 0.004)ª*	0.001 (-0.001, 0.003)
# Times a week exercising at follow-up for more than 20 minutes		*0.002 (0.001, 0.003)ª*	*0.002 (0.001, 0.003)¶*
HR-QOL at baseline		*0.174 (0.140, 0.208)§*	*0.151 (0.116, 0.187)ª*
Sex			
Females versus Males			*-0.015 (-0.029, -0.001)¶*
Age			
≥ 75 versus 65 – 74 years			-0.005 (-0.019, 0.010)
Marital status			
Married versus Not Married			-0.001 (-0.016, 0.0133)
Education			
Primary versus High School and post-secondary			0.018 (-0.006, 0.041)
Yearly Household Income			
≥ $55,000 versus < $55,000			-0.014 (-0.071, 0.044)
Area of residence			
Low/Medium versus High deprivation			<0.0001 (-0.013, 0.013)
Current smoker			
Yes versus No			*-0.045 (-0.071, -0.0198)ª*
At least one alcoholic drink, ≥ twice a week, every week in last 6 months			
Yes versus No			0.013 (-0.001, 0.027)
# of chronic physical disorders			
≥ 3 versus 0-2			*-0.022 (-0.037, -0.008)§*
Depression			
Yes versus No			-0.007 (-0.036, 0.021)
Social support			
3 versus 0-2			0.011 (-0.008, 0.030)
Number of Daily Hassles			
≥ 5 versus 0-4			*-0.022 (-0.035, -0.008)§*
R^2^ adjusted	0.01	0.12	0.15

*Estimates adjusted for all other variables in model

ª*P*<0.001

§*P*<0.002

¶ <0.05

Additional analyses looking at the change in exercise, between baseline and follow-up, showed that participants reporting never exercising and those reporting a decrease in exercise had on average a lower HR-QOL at follow-up, when controlling for all other study variables (Table 3). The results also showed that females reported lower HR-QOL on average than males. Age and area of residence socioeconomic status were not associated with HR-QOL.

**Table 3:** Change in exercise frequency between baseline and follow-up on health related quality of life at follow-up

	Health-related quality of life (EQ. 5D scores)	
	Unadjusted	Adjusted*
	Beta estimates (95% CI)*	
Change in physical activity at follow-up from baseline		
Never exercised	*−0.075 (− 0.096, − 0.053)ª*	*−0.040 (− 0.061, − 0.018)ª*
Decrease in exercise	− 0.016 (− 0.032, 0.001)	*− 0.019 (− 0.036, − 0.003)¶*
No change in exercise	− 0.002 (− 0.022, 0.017)	− 0.017 (− 0.036, 0.003)
Increase in exercise	**Reference**	**Reference**
R^2^ adjusted	0.05	0.16

*Estimates adjusted for exercise at baseline and all other study variables

ª*P*<0.001

¶ <0.05

## Discussion

This longitudinal cohort study adds to the present literature by reporting on the estimated effect of physical activity on the HR-QOL assessed at two time points, 3 years apart, in a large sample of community living older adults consulting in primary care, while also considering a number of important confounders.

In this study, exercise at baseline was a significant predictor of HR-QOL 3 years later in univariate analyses. However, multivariate analyses suggested that it was not an independent predictor of HR-QOL. The literature is not consistent on the effect of exercise on long-term HR-QOL and health outcomes. Contrary to our results, leisure time physical activity significantly predicted long-term HR-QOL as measured with the SF-36 questionnaire among older adults in a Spanish cohort study followed from 2003 to 2009, [44]. In an Australian longitudinal study of older women with a history of depressive symptoms, Heech et al., (2016) showed that physical activity measured in 2002, 2005 and 2008 only marginally predicted HR-QOL 3-years later [19]. Although both these studies controlled for a number of socio-demographic, health status factors at baseline, the analyses did not control for physical activity at follow-up.

Exercise at follow-up in our study was significantly associated with HR-QOL at follow-up. Consistent with our results, Heech et al.’s (2016) study also showed that cross-sectionally physical activity was associated with HR-QOL [19]. Similarly, a recent Canadian study, conducted in a large sample of community living older adults aged 70 years and over recruited between 2003 and 2005, showed that past physical activity during adolescence, early adulthood and mid-life, was not associated with present health outcomes (i.e. blood glucose, blood pressure, body mass index), but that current physical activity was [12].

Our results also suggested an association between reported changes in exercise habits, from baseline to follow-up, and HR-QOL. The results showed that, as compared to participants reporting an increase in how many times a week they exercised for more than 20 min*,* those remaining inactive and those reporting a decrease in exercise frequency had on average a lower HR-QOL at follow-up. Participants who maintained their exercise frequency levels reported similar scores in HR-QOL at follow-up as those having increased their exercise frequency. These results are similar to a 7-year longitudinal study, of British women aged between 60 and 79 years, by Choi et al., (2013) who reported that women increasing their activity level had increased odds of maintaining or improving their HR-QOL, while those who decreased their activity level saw a deterioration in their HR-QOL assessed with the EQ-5D [45].

In this study, we did not find a significant interaction between exercise frequency and sex. The literature on sex and gender differences in physical activity on health related outcomes are scarce. In our study, females reported on average a lower HR-QOL at follow-up and this was independent of baseline factors. We also did not find a significant interaction between exercise frequency and social and material deprivation of area of residence. Kamphuis et al., (2009) also explain that individuals living in more disadvantaged neighborhoods are exposed to more environmental concerns such as safety and this may negatively impact their perception of physical activity [15]. Although the administrative health region in which our study population was recruited is representative of urban, rural and metropolitan regions of the province [46], it is more likely that participants living close to primary care clinics may feel a greater sense of neighborhood security independent of deprivation. Dogra et al., (2015) also looked at the association between aging expectations and physical activity on HR-QOL in older adults with low socio-economic status and found that expectations regarding ageing were associated with physical activity and HR-QOL outcomes [47]. More research is needed on the gender specific associations between physical activity and health outcomes in older adults to better understand and discriminate between potential moderating and mediating psycho-social factors of this association.

This study includes some limitations. For example, our sample may not be generalizable to the general older adult population since the participants were recruited in primary care settings and may therefore exhibit more health problems than the norm, and also because those retained over 3 years differed from those lost to follow-up. Further, physical activity was self-reported and this may have been subject to a recall and social desirability bias, although these errors likely do not differ across levels of HR-QOL. Finally, although HR-QOL was not directly assessed with the original version of the EQ-5D-3 L, this should not have an effect on results since the same methodology was used in both surveys.

## Conclusion

In summary, the findings of this study show that HR-QOL decreases with time in older adults. Maintaining and increasing physical activity throughout the years in older adults however can mitigate this decrease. Future research should focus on potential moderating and mediating psycho-social barriers and the presence of physical comorbidities associated with exercising in older age populations. This will help in identifying vulnerable populations that may benefit from exercising.

## Abbreviations

EQ-5D-3 L: EuroQol-5 dimensions, 3 levels questionnaire
HR-QOL: Health related quality of life
